# Developing a comprehensive curriculum program for nurse practitioners delivering primary care in the long-term care setting

**DOI:** 10.1177/08404704241259900

**Published:** 2024-08-28

**Authors:** Adhiba Nilormi, Carrier Heer, Erin Ziegler

**Affiliations:** 1 Bruyère Research Institute, Ottawa, Ontario, Canada.; 2 26622 Brant Community Healthcare System, Brantford, Ontario, Canada.; 3 7984 Toronto Metropolitan University, Toronto, Ontario, Canada.

## Abstract

In the Long-Term Care (LTC) setting, comprehensive primary care is often provided by Nurse Practitioners (NPs). NPs are uniquely positioned to meet the evolving primary care needs of LTC residents. However, caring for this population requires additional education and training due to its special considerations. To meet the learning needs of NPs entering the LTC workplace, a Certificate Program was designed to enhance primary care competencies within the LTC setting. The aim of the program is to increase knowledge, capacity, and confidence of NPs to deliver quality, evidence-based, integrated, and interprofessional primary care to LTC residents. This curriculum is anticipated to address the growing need for LTC services and improve the delivery of high-quality primary care.

## Introduction

Delivering primary care in a Long-Term Care (LTC) setting, the role of Nurse Practitioners (NPs) is multi-faceted. NPs are clinicians, case managers, educators, and collaborators. NPs are advanced practice registered nurses with graduate education and training, enabling them to practice as autonomous healthcare professionals.^1^ NPs are integral to the healthcare system, possessing the skills to provide a wide range of primary and specialized care services.^2^ NPs diagnose and treat acute and chronic illnesses, provide preventive care, and prescribe medications.^3^ Their scope of practice also focuses on health promotion, disease prevention, health education, and incorporates leadership and research competencies.^4^

NPs work in diverse settings such as family practice and primary care clinics, hospitals, community health centres, NP-led clinics, long-term care, and home and community care.^3^ Among their diverse practice settings, NPs play a significant role in LTC, where residents are increasingly presenting with complex health conditions amidst ongoing health workforce challenges, including understaffing and high leadership turnover.^2,5^ Data indicates that LTC residents have a high burden of frailty, mental health conditions, and chronic diseases such as hypertension, cardiovascular diseases, dementia, and diabetes requiring an interprofessional, integrated approach to care.^2,6,7^ To address these challenges, governments and healthcare organizations are increasingly relying on NPs to bridge the gap in LTC services. For example, in 2022, the Ontario government announced an investment of $57.6 million over the next 3 years to recruit and retain 225 additional NPs in LTC.^8^

It is important to recognize that LTC constitutes primary care within a specialized setting. In LTC, NPs deliver comprehensive primary care encompassing routine and preventative care services such as health screenings, vaccinations, and health education as well as acute and chronic disease management.^2^ This approach is geared toward enhancing the overall physical, emotional, and social well-being of residents. Both primary care and LTC emphasize a wholistic approach to care and prioritize patient-centred practices. NPs play an important role in ensuring residents receive consistent and coordinated healthcare services including appropriate care transitions.^2^ As the ageing population increases the demand for LTC services, NPs are uniquely positioned to address the evolving primary care needs of residents. While primary care NP programs typically cover a broad spectrum of patient populations, LTC requires additional education and training due to its special considerations.

NPs have repeatedly demonstrated their value in LTC, contributing to the delivery of high-quality resident care,^9–11^ including improved resident and family satisfaction with medical services and enhanced levels of meeting care goals.^9^ In fact, a systematic review looking at the impact of NPs in LTC settings has shown that facilities with NPs on staff experience lower rates of depression, urinary incontinence, pressure ulcers, restraint use, and aggressive behaviours among residents.^9^ NPs in LTC also bring benefits such as decreased unnecessary hospitalizations,^12^ increased healthcare accessibility,^12^ optimized use of healthcare resources leading to cost savings,^13,14^ and easing pressure on the broader healthcare system.^11,13,15^ These benefits were particularly evident during the pandemic where NPs stepped in to resolve exposed deficiencies in LTC around staffing, inefficient infection control and prevention practices, burnout and turnover, providing leadership and staff training through education, and interprofessional collaboration.^16–18^

Despite recognizing the significance of NPs in LTC, there remains an existing need to develop innovative programs to educate and equip NPs to confidently navigate LTC settings.^19^ Current NP education is limited in the provision of primary care for this specialized population and many practicing NPs do not feel capable or confident to work in LTC without additional education. This article describes the development process of a Certificate Program specifically designed to enhance the competencies of NPs delivering primary care in LTC settings.

## Objectives

The objective of this Certificate Program is to increase knowledge, capacity, and confidence of NPs to deliver quality, evidence-based, integrated, and interprofessional primary care to LTC residents.

In doing so, the curriculum is anticipated to have indirect impacts including reducing healthcare costs, minimizing unnecessary transfers and hospital admissions, identifying knowledge and skills to support LTC staff capacity building, supporting with NP role clarity, improved job satisfaction and retention, and enhancing LTC resident health outcomes and satisfaction.

## Curriculum development process

Curriculum development follows an iterative process encompassing various steps, including needs assessment, curriculum outline/structure, content development, and thorough review. These steps are interconnected and aim to organize the material to be taught, identify the target audience, and determine the most effective instructional methods.^20^ This process requires a good understanding of the target audience, their professional scope of practice, and the specific needs pertaining to LTC practice.

A comprehensive needs assessment, which includes reviewing literature, gathering insights from stakeholders, and analyzing existing educational programs, is crucial to identify the gaps and learning needs of NPs and NP students in LTC.^20^ Subsequently, the process involves clearly defining learning objectives that align with the identified needs and developing a curriculum framework that adheres to practice competencies, educational standards, regulatory requirements, and best practices for NPs.

One key aspect of the curriculum development process is interprofessional collaboration. Research indicates that there is a shift in health professions education to a greater emphasis on interprofessional practice and education.^21,22^ NPs in their practice settings, especially in LTC, are part of an interprofessional team reflective of different roles and practices. A key competency of NP practice is consultation and collaboration,^4^ and research in the LTC setting indicates that NPs play an important role in enabling interprofessional collaboration and care.^23^

Interprofessional education and collaboration should not only be reflected within curriculum content for NPs practicing in LTC but also in the curriculum development process itself. Therefore, it is crucial to engage and ensure collaboration with diverse subject matter experts, stakeholders, educators, and primary care practitioners with expertise in LTC early in the curriculum planning and development phase. This ensures that the curriculum developed reflects current practice standards for NPs, interdisciplinary perspectives, and best practices.

### Needs assessment

NPs play a crucial role in LTC, yet research indicates a declining number of NPs working and/or interested to work in this area.^24–26^ There are several barriers to integration of the NP role within LTC settings, both at the organizational and individual levels. The Pan-Canadian NP Retention & Recruitment Project has identified several organizational level barriers to the optimal use and integration of NPs in practice settings, including LTC. These barriers include government regulatory and policy barriers, funding issues, lack of understanding of the NP role and scope of practice, challenges in the uptake and integration of the NP role at the organizational level, and opposition from specific stakeholders.^27^ The 2017-2018 Pan-Canadian NP Survey revealed that a significant portion (30%) of NPs working in LTC settings reported not working to their full scope of practice.^27^ Additionally, the survey indicated that LTC settings often lack adequate funding models to support the effective implementation of NPs.^27^

On the individual level, barriers include a reported lack of confidence and knowledge in caring for older adults and insufficient geriatric training within current curricula.^28–30^ A survey assessing NPs’ confidence and knowledge regarding older adult care, including in LTC settings, revealed that 95% of respondents expressed a need for specialized education in this area as part of their NP training.^31^ Another study indicated that over 60% of NPs working in LTC reported a lack of confidence in providing specialized care such as palliative and end-of-life care, and managing complex residents, prior to receiving continuing education specific to these skills.^32^ This is due to having limited exposure to content around LTC practice and specialized care, such as palliative care in their NP graduate education programming.^32,33^

Existing educational programs focusing on the care of older adults often target Registered Nurses (RNs), physicians, medical residents, or are geared toward interprofessional teams.^34–38^ An example includes the Geriatric Foundations eLearning program, developed by Regional Geriatric Program Central and accredited by McMaster University.^36^ This program is intended for a diverse range of healthcare providers such as physician assistants, medical residents, RNs, NPs, physiotherapists, pharmacists, social workers, and others involved in the care of older adults.^36^ A review of the literature indicates that few options are specifically tailored for NPs and address NP competencies. No educational programs targeted for NPs specifically address long-term care settings; most are generalized geriatric programs.^39–42^ Our curriculum is unique with its focus on the LTC setting and targeted to NPs, effectively filling important gaps in existing programming.

### Curriculum framework

**Figure 1.**
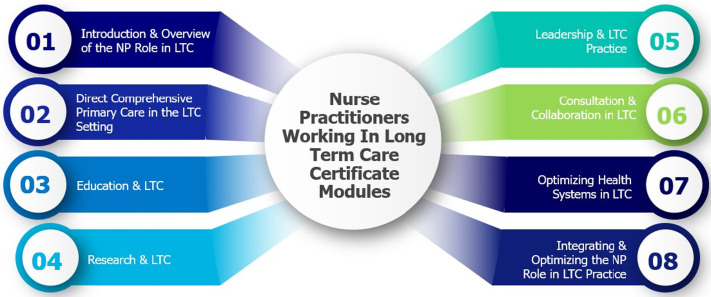
Certificate program for nurse practitioners delivering primary care in the long-term care setting: Curriculum module.

Our curriculum is developed around the Pan-Canadian Advanced Practice Nursing (APN) framework^4^ focusing on NP competencies that include direct clinical care, education, research, leadership, consultation, collaboration, and optimizing health systems, tailored specifically to LTC practice. Comprising eight modules, each module delves into these competencies, with clearly defined learning objectives (Figure 1). The selection of topics within modules underwent an iterative refinement process, involving collaboration with the project team consisting of NPs, nurse scientists, and provincial leadership specializing in LTC and geriatric curriculum development, ensuring comprehensive coverage of important topics.

**Figure 2.**
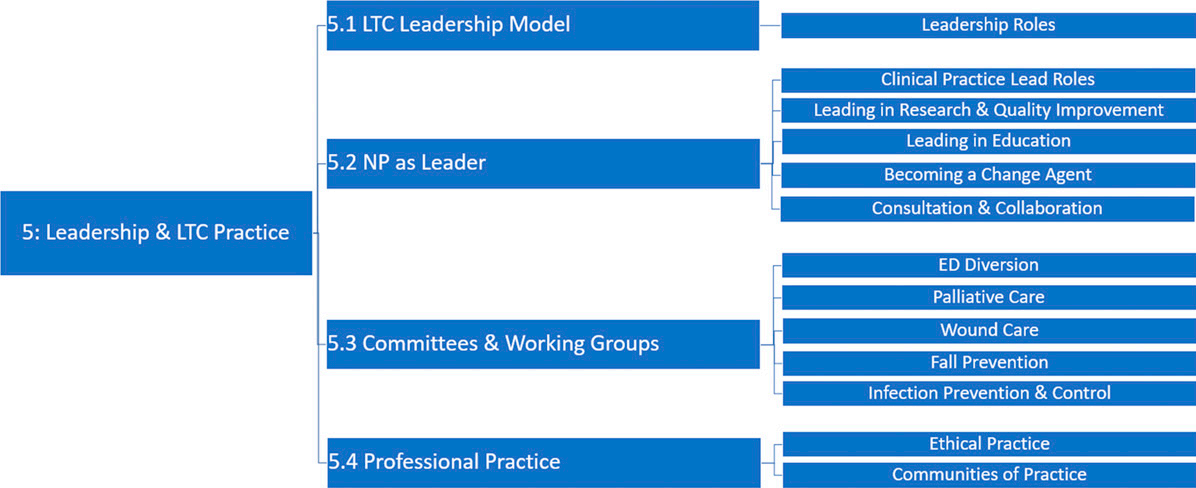
Module 5: Leadership and LTC practice.

For example, Module 5 focuses on leadership in the LTC setting, covering submodules such as LTC leadership model, NP as leader, committees and working groups, and professional practice (Figure 2).

By strategically structuring the curriculum around the APN framework and NP competencies, it aims to build upon the existing knowledge, skill, and expertise of a NP while providing speciality knowledge to support in developing, implementing, sustaining, and evaluating the NP role in LTC. Ensuring alignment with the core competencies is essential to ensure that the curriculum remains highly relevant and applicable to the NP scope of practice. This facilitates the standardization of NP training in LTC, as these competencies are nationally recognized, thereby ensuring compliance with regulatory and accreditation standards. Ultimately, this enhances the credibility and recognition of the program.

The curriculum also integrates guiding principles such as interprofessional collaboration, leadership, equity, diversity, inclusion, truth and reconciliation, psychological health and safety, and social accountability.^43^ The LEADS model of leadership is reflected throughout the curricula, specifically within Module 5: Leadership. Integration of these aspects are important to enable NPs and NP students to promote wholistic care, foster cultural competence in an increasingly diverse population of residents and staff in LTC settings, effectively address healthcare disparities, enhance mental health support and retention of NPs, and encourage social advocacy and responsibility.

The curriculum offers approximately 45 hours of self-directed and asynchronous learning. Each module includes self-directed and reflective learning exercises, recorded lectures, videos, additional readings/resources, and pre- and post-test evaluations. Case studies, referenced as resident examples within the curriculum, have also been included on acute/episodic illnesses, chronic disease and pain management, palliative and end-of-life care, leadership, consultation and collaboration, and optimizing health system in LTC. Overall, these features support the diverse learning needs of students.

### Content development

We initiated the content development process by compiling existing resources and materials relevant to each module’s topics. These resources served as a guide for developers and were included as additional resources in the curriculum to supplement student learning. An interdisciplinary team of 17 NPs, RNs, and nurse scientists with geriatric, LTC, and academic expertise and practice was engaged to develop content. This included an Indigenous NP to develop topics related to truth and reconciliation and Indigenous nurses’ engagement.

Content developers with the requisite expertise were identified and engaged via leveraging existing informal and formal professional networks, communities of practice, collaborative tables, and provincial tables. A matching process ensued to align developers’ areas of expertise with the curriculum topics. Throughout the development process, developers received guidance and feedback to ensure the academic rigour and relevance of the content, with a focus on utilizing Canadian sources to ensure applicability nationwide.

### Curriculum review

The curriculum underwent a comprehensive review process to ensure alignment with overarching objectives and specific module goals, as well as adherence to the five guiding principles. The review consisted of two rounds: a module-by-module review followed by a final full curriculum review. Interdisciplinary reviewers, including NPs, provincial geriatric leaders, and physicians practicing in LTC, were selected based on their expertise. In the initial round, each module was evaluated by two external reviewers using a detailed checklist to provide feedback. Reviewers assessed the modules for content quality, identified areas for improvement, and provided recommendations. Feedback was deliberated upon by the project team to validate recommendations and implement necessary revisions. Subsequently, two expert reviewers experienced in curriculum development and evaluation conducted a final review of the entire curriculum. Their feedback was incorporated to ensure the curriculum’s readiness for implementation.

## Key lessons learned for broader spread and scale

There are some key lessons learnt in this project. Firstly, the curriculum development process is rigorous and iterative. It requires strategic planning and innovative and critical thinking skills. It also requires organizational skills and effective coordination among the project team, content developers, and reviewers. Secondly, successful curriculum development necessitates collaborative efforts among stakeholders with diverse clinical, academic, and curriculum development expertise. Challenges can arise when there is a lack of prior educational curriculum development experience. Therefore, it is important that the content is vetted and peer-reviewed by a diverse group of primary care practitioners and provincial geriatric leadership experts. Effective communication and feedback are also key to ensure that the content developed meets the expectations and is aligned with the objectives of the program. Lastly, acknowledging the constraints in terms of time, budget, and human resources is crucial. The curriculum development process and ensuring sustainability requires careful allocation and optimization of available resources.

## Future direction/conclusion

The final curriculum is presented in an open access pressbook format that can be easily distributed, disseminated, and integrated into existing NP academic programs and organizations supporting continuing education and NP professional development. This approach will enable broader dissemination and potential credentialling opportunities across various institutions nationally. Collaboration with a diverse range of partners to develop this transformative curriculum also provides an opportunity for future dissemination within existing educational programs or the creation of new programs to meet the demand for expanded LTC NP education.

The development of this 45-hour curriculum program aims to offer sufficient educational preparation and a comprehensive overview of relevant subject matter for NPs already working in LTC settings or those interested in entering this field. It also provides additional resources and guidance for delving deeper into specific sub-topics of interest. In doing so, this curriculum is anticipated to address the growing need for LTC services and improve the delivery of high-quality care.

Moving forward, it is important to engage in future research endeavours, seek additional resources, and establish partnerships with organizations nationwide that share an interest in advancing interprofessional collaborative primary care in LTC. These efforts will play a crucial role in ensuring the widespread adoption and scalability of this curriculum on a national level.

However, it is important to note that evaluating the practical implementation and uptake of the curriculum among NPs and NP students was out of the scope of this project. Therefore, further research is required to assess changes in knowledge, confidence, competence, and the impact of learning outcomes on practice and the quality of care provided in LTC settings. To undertake evaluation research and assess the curriculum’s effectiveness in achieving its intended direct and indirect outcomes/objectives, further and sustained funding and resources will be essential to maintain a robust evaluative component.
